# Lay perceptions of diabetes mellitus and prevention costs and benefits among adults undiagnosed with the condition in Singapore: a qualitative study

**DOI:** 10.1186/s12889-022-14020-z

**Published:** 2022-08-20

**Authors:** Jumana Hashim, Helen Elizabeth Smith, E Shyong Tai, Huso Yi

**Affiliations:** 1 Saw Swee Hock School of Public Health, National University of Singapore, and National University Health System, Singapore, Singapore; 2 Family Medicine and Primary Care, Lee Kong Chian School of Medicine, Nanyang Technological University Singapore, Singapore, Singapore; 3 Division of Endocrinology, University Medicine Cluster, National University Hospital, Singapore, Singapore

**Keywords:** Risk perception, Health communication, Type 2 diabetes, Qualitative study, Singapore

## Abstract

**Background:**

Therapeutic lifestyle changes can reduce individual risk of type 2 diabetes (T2D) by up to 58%. In Singapore, rates of preventive practices were low, despite a high level of knowledge and awareness of T2D risk and prevention. The study explored the context of the discrepancy between knowledge and practices in T2D prevention among adults undiagnosed with the condition.

**Methods:**

In-depth interviews with 41 adults explored lay beliefs of T2D and the sources of these perceptions, subjective interpretation of how T2D may impact lives, and perceived costs and benefits of practising preventative behaviours. Purposive sampling was used to maximise the variability of participants in demographic characteristics. Thematic analysis was conducted to identify themes related to the domains of inquiry.

**Results:**

Participants’ risk perceptions were influenced by familial, social, and cultural contexts of the representation and management of T2D conditions. The adverse effects of T2D were often narrated in food culture. The cost of adopting a healthy diet was perceived at a high cost of life pleasure derived from food consumption and social interactions. Inconveniences, loss of social functions, dependency and distress were the themes related to T2D management. Participants’ motivation to preventive practices, such as exercise and weight loss, were influenced by short-term observable benefits.

**Conclusions:**

T2D risk communication needs to be addressed in emotionally impactful and interpersonally salient ways to increase the urgency to adopt preventative behaviours. Shifting perceived benefits from long-term disease prevention to short-term observable wellbeing could reduce the response cost of healthy eating.

**Supplementary Information:**

The online version contains supplementary material available at 10.1186/s12889-022-14020-z.

## Introduction

Globally, 1 in 11 adults live with diabetes, and 10% of health expenditure is spent on treating individuals with diabetes [1]. Complications from type 2 diabetes (T2D) like renal, ocular, cardiovascular disease, and lower extremity amputations can lead to premature death and loss of productivity among the working-age population. In Singapore, the prevalence of T2D is projected to be 15% overall and about 40% of those over 60 years in 2050 [2]. The economic burden is expected to increase to SG$ 2.5 billion by 2050 [3]. However, about 80% of T2D incidences can be prevented by reducing modifiable risk factors with lifestyle changes [4]. Preventive behaviours, including weight management, physical activity, and healthy eating can reduce one’s risk for diabetes by up to 58% [5–7].

Since the Ministry of Health in Singapore declared a “War on Diabetes” in 2016, the Health Promotion Board (HPB) launched a myriad of prevention efforts like the ‘National Steps Challenge’ to increase physical activity and ‘National Diabetes Reference Materials’ to disseminate information on risk management [8]. While these efforts may have increased awareness and knowledge, practice of preventative behaviours is still suboptimal. Nationally, 81% had adequate functional health literacy regarding diabetes [9]. However, the 2020 national population health survey found an increase in obesity prevalence across all age groups compared to 2017 [10]. A household survey in 2019 found that 86% and 89% of participants agreed that healthy eating and exercise respectively, can control the risk of diabetes. However, only 37% ate the recommended 5 servings of fruits and vegetables, and 28% met HPB’s physical activity recommendation of 150 min/week [11]. To reach higher engagement in T2D prevention, the discrepancy between knowledge and practice needs to be addressed.

In addition to knowledge, contextual and individual barriers can influence behaviour. While accessibility challenges due to logistical or financial reasons and psychological barriers due to limited perceived benefit or threat were identified to influence T2D prevention across 12 studies globally, the expressed reasons were diverse, complex, and context-specific [12]. The influence of culture and social environment on the likelihood of undertaking preventative measures highlights the importance of understanding subjective experiences and values. Lay beliefs are the subjective and informal ways individuals contextualise their actions, affecting motivation to adopt healthy and preventative behaviours [13]. Understanding lay beliefs and risk perceptions in context would help explain why knowledge does not translate into practice and identify intervention focal points to increase the effectiveness of current T2D prevention efforts. In this study, we qualitatively explored the lay beliefs and risk perceptions of T2D, and attitudes towards preventive behaviours among adults undiagnosed with the condition.

## Methods

### Participants and procedure

The study population was adults aged between 30 and 60 years without a diagnosis of T2D. Our sampling strategy ensured the diversity of the ethnic groups as T2D was more prevalent among people with Malay and Indian background than Chinese background [2]. A study invitation was posted on bulletin boards in primary care clinics and circulated through email and social media like Facebook. During the screening of eligibility, we collected age, ethnicity, education level, and housing to ensure our recruited participant sample represented the Singapore population by demographic. We had an overwhelming interest from Chinese participants and a few Malay and Indian participants. To address this, we encouraged recruited participants to circulate the poster to their Malay and Indian friends. Interviews were conducted on a video conference call or in-person and in English, Mandarin, Malay, or Tamil. We employed several approaches to ensure the consistency of data collection in Chinese, Malay, and Tamil. We made an interview protocol describing the details of interview data collection: preparation, informed consent, introduction, a topic guide with probing questions (see supplementary file), debriefing, and rules of translation and transcription. Researchers who conducted the interviews in Mandarin, Malay, and Tamil were appropriately briefed by the first author (JH), who conducted majority of the other interviews in English. JH has training in behavioural science and qualitative research. Additionally, 2 of the 3 of these researchers were current PhD candidates with focus on qualitative research. The third researcher went through an extensive training and several practice rounds with JH before they independently conducted the interviews.

Interviews lasted from 40 to 70 min. Participants were provided with a voucher of SG$30 for compensation for their time. With 41 participants, our interviews had reached theoretical and thematic saturation. Theoretical and thematic saturation was determined using a hybrid and an iterative process using three criteria, as discussed by JH and the last author (HY), who is an expert in qualitative research and health psychology: (1) holistic understanding of each emergent theme to illustrate them appropriately, (2) three consecutive transcripts with no new themes found and (3) sufficiently diverse range of perspective, when our study sample relatively represented the Singapore population [14–16]. The study was approved by the university research ethics committee. All methods were performed in accordance with the ethics committee’s guidelines and regulations and the Declaration of Helsinki.

### Interview guide

We sought to identify the social and cultural attributes that participants considered when thinking about T2D and how they may contribute in weighing the ‘cost’ and ‘benefit’ of engaging in T2D prevention. An interview topic guide was developed based on the literature review on knowledge, attitudes, and practice of T2D prevention with a focus on three major domains: (1) lay beliefs of T2D and the sources of these perceptions; (2) subjective interpretation of how individuals thought T2D might impact their lives; and (3) perceived costs and benefits of practising preventative behaviours. The interview topic guide is provided as supplementary material.

### Analysis

Interviews were audio-recorded, translated for non-English interviews, transcribed verbatim, and entered in NVivo 12. Non-English interviews were read by JH and HY after transcription to ensure there was consistency in data collection across the different researchers before entered into the software. The six-phase reflexive thematic approach was used for analysis [17]. JH and HY read the transcripts while interviewing to familiarise the data and revise probes if necessary. After the first 10 interviews, JH and HY worked independently before discussing the preliminary codes and generating initial themes. The initial code scheme was applied to the remaining interviews using a constant comparison method to refine and finalise the codebook. This also allowed us to assess theoretical and thematic saturation. We addressed reflexivity throughout the phase of data collection and interpretation to check any influence of preconceptions and ensure that significant findings were not left out or misinterpreted. The interviewers had regular meetings for debriefing and checking whether personal attributes, qualifications, experiences, and values affect interviewing and leading interview questions. The second author (HES) is an expert in primary care and community engagement; the third author (TES) is an endocrinologist and an expert in diabetes prevention, especially in Singapore. All had regular meetings where the first author presented preliminary data analysis, and all discussed and finalised data interpretation. To ensure the credibility of the analysis, a subset of the participants (*n* = 8) was invited to a workshop to discuss the findings as a form of member-checking. They were selected based on the following criteria: (1) participants had consented to re-contact during informed consent, and (2) participants were available for a focus group discussion, which allowed the participants to freely talk among themselves as we took overall notes. This was not part of the data collection; therefore, we did not record or transcribe their discussions. No major change was made as the consensus was positive and in agreement with the themes we identified. We reviewed their comments after member checking to iterate and strengthen our themes and the narrative to weave them together.

## Results

Table 1 shows the demographic characteristics of 41 participants. There were 24 females and 17 males, with 16 participants in their 30 s, 14 in their 40 s, and 11 in their 50 s. Ethnic distribution followed 61%, 15%, and 20% for Chinese, Malay, and Indian, respectively.

**Table 1:** Characteristics of Participants

	Study Participants (*N* = 41)		Singapore Pop
	N	%	%
Age			
30–40 years	16	39%	33%
41–50 years	14	34%	34%
51–60 years	11	27%	33%
Gender			
Male	17	41%	48%
Female	24	59%	52%
Ethnicity			
Chinese	25	61%	75%
Malay	6	15%	12%
Indian	8	20%	10%
Others	2	5%	3%
Education			
≤ Secondary School	13	32%	40%
Post-secondary	12	29%	26%
≥ University	16	39%	35%
Housing			
≤ 3-room HDB flat	11	27%	24%
4- to 5-room HDB flat	23	56%	55%
Condominium or Maisonette	5	12%	16%
Private Property	2	5%	5%

*HDB* Housing Development Board; Singapore’s public housing scheme

Table 2 presents a hierarchal thematic scheme of the novel findings. We identified 5 main themes, each with 3 sub-themes: (i) perceptions of diabetes, (ii) sources of perceptions, (iii) relational identity between food and T2D, (iv) perceived losses from healthy eating in T2D, and (v) perceived gains from physical activity in T2D. Even though the findings are categorized by the domains of inquiry, all the sub-themes are interrelated and create the narrative of the given context.

**Table 2:** Hierarchical Thematic Scheme

Categories/Themes	Sub-Themes
**Lay beliefs of T2D**	
Perceptions of diabetes	Initial stages of T2D create inconvenience to lifestyle
	Complications from T2D can impact QoL
	Progression of diabetes is slow
Source of perceptions	Expert knowledge has limited influence
	Influence of the media’s portrayal of characters with diabetes
	Personal encounters with people living with T2D
**Subjective interpretation of how T2D impact lives**	
Relational identity between food and T2D	Perceived susceptibility directly correlated to sugary food intake
	Restriction of diet in relation to Singapore’s food culture
	Convenience and cost of unhealthy vs healthy food
**Practicing preventative behaviours**	
Perceived losses from healthy eating in T2D prevention	Interaction with food during gatherings and celebrations
	Pleasure derived from food consumption
	Influence of food on social identity
Perceived gains from physical activity in T2D prevention	Exercise desirable despite challenges
	Immediate observable benefits of exercise
	Self-improvement by tracking progress

*QoL:* Quality of Life, *T2D:* Type 2 Diabetes

### Perceptions of diabetes

All the participants were aware of diabetes with a good understanding of its risk factors, like obesity, family history, dietary habits, and sedentary lifestyle. Commonly cited symptoms included increased thirst, frequent urination, changes in weight, and “sweet pee which attracted ants.” A few responded there would be no visual symptoms until a blood test has been taken. Participants also had a good understanding of the disease progression. Apart from the cost of treatment, the initial stages of the disease management were perceived as “inconvenient” due to the daily medications and diet considerations.

*If I had it, I had to take medications regularly and properly. I had to bring medications with me. It’s inconvenient. If I were to be in a social setting, I’d be like, “Oh, I’m sorry, I can’t eat this or drink that” or like “I need to take my medication.” Then, people would look at me weirdly. I’d be like, “Should I explain or not?” (30s, F, Chinese)*

Later stages of T2D were perceived as “disastrous to quality life” due to the complications arising from T2D. Many participants were concerned that they may become a burden and be unable to care for others. Complications of T2D were associated with disabilities that could cause “(loss of) ability to work, (and) ability to live independently.”

*There is a risk of complications like having kidney problems, amputations, or maybe even blindness, or losing your sensitivity, your extremities. These are the complications that someone with diabetes will have to anticipate. But if I develop complications that result in me developing blindness or limb amputation, that one will be quite disastrous to the quality of my life. (30s, M, Chinese)*

However, most participants expressed that the development of these complications would be far away, and the progression from the initial stage to complications would be slow. They believed such a slower progression of diabetes compared to other diseases meant that it was not as life-threatening and that diabetic patients have an opportunity to control and manage diabetes with medication and lifestyle adjustment.

*You may have diabetes, but it may not happen like a one-shot. For diabetes, first, you have medication to manage it. You have time for treatment. You still can control in a way. You can try to minimise potential injuries. It will not get fatal as compared to heart disease where it strikes up, the recovery time and saving the person is very acute (30s, M, Chinese)*

### Sources of perceptions

Participants said they actively seek “expert” knowledge only after specific triggers like health screening results or hearing about T2D diagnosis from their social circles. Some participants found the amount of information and use of jargon overwhelming, and the information on actionable steps sometimes contradicting.

*I usually inquire into a condition when somebody I know is diagnosed with the condition. It usually takes a few searches to understand because there are many sources, which tend to be overly clinical in their jargon, which is not very helpful and only targeted to medical professionals. Usually, the contradictions are not in the diagnosis but understanding if it is major or minor, or if any meaningful action should be taken. (50s, M, Chinese)*

Hence, many lay perceptions were influenced by the media portrayal of diabetic patients. Participants recollected that the characters with diabetes in the media were often in the later stages with limb amputations, which were somewhat disturbing. However, diabetes was rarely reported as a cause of death, even if it was an underlying health condition.

*To me, diabetes is a bit far away. We hear about stroke and heart attacks when the media reports that ‘somebody collapsed while jogging’. Whereas, when somebody dies from diabetes, we don’t usually read it in the papers. You might die of heart attack with a pre-existing condition of diabetes. But people just report your heart attack. Diabetes tends to be at the back of everybody’s mind. It exists, but the media doesn’t put it in the spotlight that often. (30s, M, Chinese)*

For participants who had family, relatives, or friends with T2D, their perceptions of the disease – cause, risk, and consequence of diabetes – were influenced by what they observed and heard from the patients. In particular, participants who had parents and relatives with late stage-associated conditions, their descriptions about the impact of T2D on life were specific and vivid.

*She suddenly started to bleed very badly after just gently scratching a black spot, but she didn’t feel any pain. She passed out at home because of the excess bleeding. We had to call the ambulance, and she had to go for another operation for her leg. When you have diabetes, it will take longer for the wounds to be healed, so it took her a long time to heal. This is a real problem. (40s, F, Indian)*

### Relational identity between food and T2D

Common factors influencing perceived risk among participants were poor health screening results, obesity, positive family history, and unhealthy practices, especially around dietary choices. Many participants perceived that having too much sugar was the main cause of diabetes, which translated to reduced perceived susceptibility of T2D among those who did not have many sugary foods.

*I think my risk is very low. I am someone who is not into sugar - I don’t drink bubble tea, I don't have a lot of sweets, biscuits or cakes or chocolates. I don’t have that kind of craving. (40s, F, Chinese)*

Several participants said when friends and families speak about diabetes, it is usually candidly referring to having too many sweet food items. However, the colloquial reference to sweet foods and sugar as the cause of diabetes did not reduce the consumption of these foods.

*When you have a gathering, you look at the amount of food and sugar. Then, you casually say like ‘this is going to get me diabetes.’ But it’s a form of a joke than anything serious. (40s, M, Malay)*

Participants were asked to share how they thought their lives could be impacted if they were to be diagnosed with T2D. A common perceived loss was related to the restriction of diet to manage T2D.

*If I had diabetes, I would have to have a more restrictive lifestyle. I would not be able to eat as much of the food that I enjoy – snacking, eating ice cream and things like this. I myself have sweet tooth. For me having to be a bit more restrictive would be quite a downer (30s, M, Chinese)*

Many participants shared that the diet restriction was particularly impactful in Singapore as the local food culture is important in shaping the Singaporean identity. With the variety of food, there were expectations of having a certain level of culinary experience during social gatherings.

*Given that we are Singaporeans, we love to eat. It is difficult to maintain a healthy lifestyle or a healthy diet. Our culture is about eating – we have a fusion of food and all kinds of foods from all around the world. Even if healthier, people do not want to meet friends over a fruit platter. They will meet for a Korean barbeque. So, from a cultural perspective, it’s very hard to disconnect from food. (30, M, Chinese)*

Participants defined good food as tasty and cheap and shared that people are willing to travel significant distances in search of good food. Singaporeans take pride in finding food that has the best value for money, and this pursuit is often a topic of conversation among friends and family.

*I think it is difficult for people to control their diet. Singaporeans like to travel around to find food to eat. They might be living in [a neighbourhood in the east], but they do not mind travelling to [a neighbourhood in the west]. They want the best food that they can get for the three dollars fifty cents. They will talk to each other about where to go and what to eat. They enjoy eating so much and want total value for money in getting the best bang for their buck. (40s, M, Indian)*

Participants pointed out the convenience, ease of access, and budget-friendly options; ‘hawker centres’ located within every public housing estate providing diverse local cuisines quickly and cheaply. Furthermore, in recent years’ options of delivery service and the availability of all types of cuisine, one can access cheaper and more delicious food any time, from the comfort of home.

*When you are craving something, or you want to eat something, usually I must travel all the way there. But now everything is a lot easier to eat something, and it will come to your doorstep. Even if I am tired or it is late at night, and I feel like having ice cream, there is [food delivery platform]. So, there are a lot more opportunities to indulge in these kinds of things. (30s, F, Chinese)*

Conversely, many participants pointed out that healthy food options are more expensive and can take a long time to prepare.

*In Singapore, the faster and cheaper options are unhealthy. So, if you want to prepare healthy food, and you have working hours, you need to make a lot of sacrifices – like wake up early or prepare it the night before. And the ingredients for healthy meals are not cheap. (40s, F, Malay)*

### Perceived losses from healthy eating in T2D prevention

When speaking of restrictive diet and healthy eating, participants alluded to a ‘loss’ in their lifestyle due to reduced enjoyment and impaired social interactions associated with food. Social relationships and celebrations are centred on food, and declining food or refusing to eat could be interpreted as an insult to the host. This was mentioned by participants across all the ethnic groups.

*You’re stopping me from eating my favourite food, you know? I rather die. What makes it really hard is that any form of Chinese celebration has got to do with food. The bigger the celebration, the more food we have. It’s like, if you don’t eat, you’re extremely rude – it’s insulting not to eat something that is placed before you. (50s, M, Chinese)*

Many participants also shared that eating provided a source of enjoyment and that some participants turned to food when they were upset or stressed. While some participants shared that they exercise to de-stress, some participants shared that they eat to de-stress. A participant mentioned the endorphins released when exercising, while another said the same but when indulging in delicious food. While there was awareness for the need to mitigate the effects of unhealthy eating, it came in the form of compromising other meals instead of giving up the pleasure derived from unhealthy foods.

*The only thing that I’m doing now to control my eating is trying not to have breakfast in the morning. I will just try to have lunch and dinner, but it is usually not controlled. I should stop eating less fried food. But I don’t think I can give up fried chicken that easily. It’s just really too good to give up. (30, M, Chinese)*

Similarly, some participants expressed that they justify their eating habits by having ‘earned their calories’ after exercising and consider their indulgence as a reward. The influence of social media culture was also reported, where people post pictures of the aesthetics of the setting and the food. Participants shared that social identity is associated with food and enjoying life, and rarely with healthy eating in the context.

*People eat to survive. But for me, I live to eat because I love to eat. So, if I’m not happy, I need to eat to be happy. I love food. To continue eating unhealthy food, I compensate for it by doing more exercise. So, I had the calories burned to eat. If I don’t exercise and eat, I’ll get fat or something like that. But if I exercise and eat, it can balance out, right? Nowadays, people post their food on social media. Wow, they’re so yummy! But, if you burn a fish at home, you wouldn’t post on social media. You will only post nice and presentable ones. (40s, F, Chinese)*

### Perceived gains from physical activity in T2D prevention

Demanding work environments and familial responsibilities created multiple competing priorities even though exercise is desirable. These responsibilities often lead to sacrificing sleep, poor eating habits, and exercise time to meet these expectations. Participants shared that Singapore’s competitive work environment creates high-stress situations. There is an expectation to constantly improve skills and qualifications to ensure job security.

*Stress is one contributing factor. People tend to eat more and badly when they have stress. People want to have job security. Now there is digital disruption, so you can become invalid, which is quite scary. So, we need to upgrade ourselves. I have attended many courses, and I will attend more, so there is no time to exercise sometimes even though I want to. (40s, M, Others)*

Participants, especially mothers, shared that time for themselves when they could exercise is seen as a “luxury” or “culturally challenging”. A Muslim woman shared how she felt different and watched when running with a hijab, a head covering worn by many Muslim women. Internalised expectations of clothing worn during exercise contrasted with wearing a hijab, creating potential psychological barriers.

*I’m making a conscious effort, but it is really tough for me to find a time with kids, work, and everything. Most of the days, as a working mom, I seldom get time for myself to do what I want. You feel good about yourself with those endorphins. It is very good to mentally detox by getting away from home and kids. But, when I ran in the park, I used to feel a bit shy and embarrassed because I was wearing hijab, and then I felt like everyone was looking at me (30s, F, Indian)*

Despite these challenges, there is interest to engage as several participants shared their rewarding experiences from physical activity. When talking about physical activity, participants alluded to a ‘gain’, citing how they feel ‘lighter’ and ‘good’ after exercising. Participants shared that initial adoption of physical activity was often in response to an external cue, including a worrying health screening result or a recent loss of life. Accountability through exercise programmes or friends or incentives were cited as facilitators of engaging in physical activity. However, the reasons to sustain behaviour were to ensure they could maintain their physical appearance, retain independence and physical mobility to continue doing the things they enjoy, or continue experiencing the immediate benefits and enjoyment of certain exercises.

*“I don’t want to be obese or unhealthy. I don’t want to inject myself all the time or spend my hard-earned money on doctors or medications. So, I exercise. Then, I’ll feel lighter. I’ll feel good, fit. I’ll be happy, and I can do a lot of things. Through all these exercises, my muscle won’t be so stiff. I can do a lot of things together with my children. I can cook for them and continue to work. Then I can go travel if money permits.” (50s. F. Chinese)*

Many participants also reported that observing self-improvement by tracking progress acted as positive feedback for their self-efficacy, and in return, motivated them to exercise further or longer. Participants who exercise regularly also pointed out that exercise is a more individual activity, and therefore it is not affected like healthy eating by its social context.

*One day you cycle down a road, you see some things and buildings. Then the next time, you motivate yourself to cycle further. It’s with running also – in my mind, I will motivate myself to jog slowly. And then now I can run to this place, to that place, and then further. Then slowly, I can run back. It motivates me. So every time, you look for a new goal to achieve. I can go somewhere further, you know? (40s, F, Chinese)*

## Discussion

The study findings highlight the importance of understanding the social and cultural contexts of T2D risk in the development of effective interventions among adults who have elevated risk yet do not engage in prevention behaviours. The findings explained the dissonance between knowledge and practice of T2D behaviours. In the study, dietary change was generally perceived negatively due to the hedonistic approach to food and its strong association with Singapore food culture and social interactions. Further, access to healthy food required more effort to prepare and costs more than unhealthy options. Visible impacts from healthy eating, like weight loss, can be small and slow, creating limited observable short-term benefits. Time needed for physical activity can also be overshadowed by competing priorities of work and familial commitments. However, exercise was perceived to have short-term gains related to wellness and physical performance. These gains contribute to the positive feedback loop and enforce self-efficacy of behaviour, making one more confident and motivated to practice. Hence, the differential view of loss and gain associated with healthy eating and exercise could influence the varied sustainability of the respective behaviours.

Our findings align with a local study that showed high awareness of diabetes and perceived efficacy of preventative behaviours and shed some light on why actual uptake of these behaviours may be low despite the high knowledge [11]. Perceived severity of diabetes comes from the downstream complications of the later stages of T2D, which seem to be distant for those without T2D. With an incomplete understanding of diabetes, the lack of sugar consumption creates a lower perceived susceptibility to T2D. The perception of T2D developing slowly and being influenced by personal experiences was also reported in a similar study in the US [18]. Temporal discounting of the future reduces the benefits of preventative behaviours especially given the short-term costs [19], and optimism bias can also lower the perceived risk of diabetes [20]. These give rise to lower levels of motivation for behaviour change and the lack of urgency to act now.

Economic utility theory, which suggests that people will only change their behaviour if the perceived benefits outweigh the perceived costs, could explain why some people might not see adapting healthy eating now to prevent T2D in the future as a worthwhile investment [21]. Perceived costs of healthy eating overlap with the perceived losses of developing diabetes; they are both associated with an increased cost of time and money. Diabetes prevention and management both require dietary changes related to impaired enjoyment and social interactions. Pleasure derived from food was so important that individuals looked to various compromises and justifications to continue eating their favourite foods. Conversely, perceived benefit is weak due to the limited observable short-term benefits, creating present bias [22]. Hence, when assessing the costs and benefits of healthy eating, there might be hesitance to pay these ‘costs’ now instead of waiting until the risk is higher, or even until diagnosed with T2D.

Physical activity, on the other hand, has important short-term benefits that create a positive feedback loop. The importance of positive feedback in diabetes prevention and management through short-term gains was deemed particularly important as altering blood glucose can be discouraging due to a lack of observable results [23]. Though physical activity shares short-term costs like time and effort with dietary change, it is less subjected to some of the barriers like impaired social interactions and pleasure. Physical activity is instead seen to facilitate social interactions and pleasure. Other facilitators include incentives and tracking progress, which aligns with the successes of the National Steps Challenge (NSC) [24] but can be challenging to leverage for healthy eating. Lack of objectivity in food measurement resists an incentive-based framework, but HPB has launched the ‘Eat, Drink, Shop Healthy Challenge’ that is currently attempting this. However, like NSC, it is likely to fall victim to compliance and sustainability issues.

Translating our findings using the constructs of Protection Motivation Theory (PMT) can inform strategies to address the dissonance between knowledge and practice. PMT suggests that threat and coping appraisals influence the intention of behaviour following an emotion-evoking stimulus [25, 26]. Our findings indicate that both the threat appraisal and coping appraisal were sub-optimal among adults without T2D in Singapore. Using an appropriate risk communication tool to return health screening results to elicit an emotional and salient response is an opportunistic time to elevate this threat appraisal. Health care professionals should be vigilant in appropriately framing the consequences of living with diabetes. In efforts to encourage active diabetes management, it was communicated that diabetes does not prevent a fulfilling life, which may have reduced the perceived severity of T2D. An elevated risk appraisal is associated with an increased intention for behaviour change due to heightened emotional response and perceived severity [27]. To increase coping appraisal, it is important to address barriers and create positive associations to preventative behaviour. Reducing perceived response cost and increasing perceived benefits through observed performance is critical for sustainable behaviour change [28].

A key systematic-level barrier cited was the accessibility of healthier food options due to increased cost and inconvenience. The implications are likely inequitable among the different socio-economic groups of the population. Health outcome inequities due to the cost of healthy eating, an example of social determinants of health, have been demonstrated globally [29–31]. Structural interventions are necessary to address health inequity, such as direct health promotion at the point of sale (e.g., labelling policies on menu boards and food packaging) and food supply interventions to support the “Healthier Hawker Program” [32]. Understanding the unique barriers of the different ethnic groups could also address the differentiated risk profiles. The tension between internalised expectations of exercise and culturally accepted practices among Muslim women has also been demonstrated among their communities in the UK [33]. Further, in-depth exploration of challenges specific to Singaporean-Malay women has uncovered similar findings to ours [34]. Even though Malays and Indians are disproportionately affected by T2D [2, 35], there are no population-tailored interventions to address this health disparity. For example, providing accessible exercise spaces for Muslim women, who are usually Malay or Indian, can promote privacy and inclusivity.

When creating positive associations, short-term benefits need not be health-focused. Highlighting benefits associated with well-being can influence motivations for uptake of T2D prevention [36, 37]. Moving to non-health but valued aspects of well-being can create salient perceived benefits and leverage present bias. This is especially beneficial among those who perceive to have little to no risk of diabetes and do not perceive the need for change [23]. For example, the Asian tendency to put familial and work responsibility above self-care is reflected in the time-management barrier. However, successfully fulfilling these responsibilities contribute to their quality of life. Hence, shifting narratives on how healthy eating and physical activity can facilitate one’s career growth and in taking care of their family not only honour what is important but can also make the perceived benefits larger than perceived costs.

Shifting narratives is not easy. Given the influence on the perception of T2D, media platforms can be an important channel to consider for such dissemination. It is important to tailor the language appropriately to avoid overwhelming amounts of medical jargon. Dramatic television series have demonstrated impact in showcasing lived experiences of diseases which can shape perceptions and attitudes [38]. However, with the younger population, leveraging culturally popular individuals may be more effective. Social media ‘influencers’ have increased uptake of healthy behaviours by associating exercise and dieting with health and happiness [39]. These channels can be particularly useful in demonstrating how healthier choices can be enjoyed and integrated into social aspects to reduce the perceived costs of dietary change.

### Limitations

We did not collect data from other stakeholders, such as healthcare providers, to understand their perspectives. Primary care physicians could provide insights on their conversations with at-risk patients to gain insight on common challenges and success stories. This could have been particularly beneficial as it addressed potential response bias. Since health-seeking individuals are more likely to participate in health research, we might not have captured the experiences of a particularly important group of population. Our recruitment strategy was reliant on social media and bulletin boards in primary care clinics which might have left out perspectives of individuals who did not engage with either of those platforms. Providing a voucher as compensation for their time could have added an incentive to share the study details with their families and peers making our study prone to selection bias. We attempted to minimise this by recruiting participants across all socio-economic groups. While all the interviewers grew up in the region and were familiar with the local jargon and speaking the same language as the participants provided a level of comfort, they were all female. Having male interviewers might have made some participants more comfortable sharing certain topics.

## Conclusions

Lay beliefs and perceptions of T2D risk are contextual, shaped by social representations of the disease conditions and cultural practices relating to T2D prevention. T2D was perceived as a disease that slowly progressed and caused inconvenience and disability but did not lead to death. Motivation to practice healthy eating was suboptimal. Participants believed that it would have the same ‘costs’ as the perceived loss of pleasure from enjoying food in social interactions, narrated as the essence of the local culture and belonged identity. Cue to initiate behaviour needs to be emotionally driven in collective contexts while sustaining behaviour is through individual positive feedback from observed short-term benefits. Future T2D prevention interventions need to emphasise the roles of lay beliefs and perceptions of the disease in practices for adults who are knowledgeable but undetermined for prevention.

## Supplementary Information

**Additional file 1.** Interview Topic Guide.

## Data Availability

The datasets used and/or analysed during the current study are available from the corresponding author on reasonable request.

## Abbreviations

T2D: Type 2 Diabetes
HPB: Health Promotion Board
NSC: National Steps Challenge
PMT: Protection Motivation Theory
