# Supplementary material for: Lay perceptions of diabetes mellitus and prevention costs and benefits among adults undiagnosed with the condition in Singapore: a qualitative study
Source: BMC Public Health. 2022 Aug 20;22:1582. doi: 10.1186/s12889-022-14020-z (PMC9392359; doi:10.1186/s12889-022-14020-z)
Supplement: Supplementary file 1 — Additional file 1. Interview Topic Guide. [file 12889_2022_14020_MOESM1_ESM.docx]

**Lay perceptions of diabetes mellitus and prevention costs and
benefits among adults undiagnosed with the disease in Singapore**

Interview Topic Guide

**Objectives**

1. To explore and identify the sources of lay understanding of type 2 diabetes (T2D)
   1. What is diabetes?
   2. What are the risk factors, symptoms, and causes of diabetes?
   3. How do people construct such “folk knowledge” of T2D?
   4. How do people make a sense of expert knowledge of T2D risks?
2. To explore the subjective interpretation of how T2D may impact their lives
   1. How do people interpret risk of T2D in the context of their daily lives?
   2. How do people weigh the “cost” and “benefit” of T2D management?
   3. How do people foresee the adverse outcomes from T2D?
   4. How do the social and cultural attributes influence 2a-2c?
3. To explore the facilitators and barriers of T2D preventive behaviours
   1. How do people make a decision of adopting T2D preventive behaviours?
   2. What do people “mostly actually” do? What are the main practices in daily life?
   3. What are challenges people face in the maintenance of preventive behaviours?
   4. How do findings from 1 and 2 influence practice of preventative behaviours?

**1**. **Explore and identify the sources of lay understanding of T2D**

*First, I’d like to talk about your understanding of type 2 diabetes.*

1. Have you heard of type 2 diabetes? What do you think it is?

   [Probe: What are some symptoms?]
   1. Can you tell me about anyone you might know diagnosed with diabetes?

[If Yes] Can you describe to me how their lives have changed because of their disease diagnosis?]

[IF NO] Can you imagine a person with diabetes; how do you think their lives are different from those who don’t have the condition? how do you think having diabetes changed their lives?

1. What kind of people do you think are most at risk of diabetes?

[Probe: Ask to describe them: race, age, body type, occupation, etc]

- 1. How do you think the risk can be controlled?
  2. How easy or difficult is it to get diabetes?
  3. What are some of the biggest risk associated with diabetes?

[Probe: What is worrying or scary about T2D?

*Now, I would like to know where you get your health information on diabetes.*

1. Where do you usually look for the health information you need?

[Probe: They may look but not find – so prompt to see if they find what they are looking for in these sources]

3.1 How do you feel about the information that you find?

[Probe: share who, what and when these information are shared. If possible, share some examples and process with them to clarify what they meant]

1. Which types of information do you get from which source?
   1. Are they contradictory or different from one another?
   2. Are there specific things that you don’t understand? How does this affect whether you take any steps?
2. Among those, what is your first choice? Why is that? Are some sources more useful than others? And if so, why?

[Probe: Do you think it’s because who said it or the way it was shared? Is it also the most memorable and impactful?

1. When you look for information on diabetes risk and management, have you ever had unanswered questions?

[Probe: Let’s try to get specific data on what exactly they are. How do they solve questions they have?]

- 1. From the information you gathered, was there any information that were different from what you thought?

[Probe: differentiate between expert vs lay knowledge. Or, any misinformation]

1. Have you ever talked about diabetes with your family members or friends?
   1. What are the common concerns with your family? With your friends?
   2. How did this conversation differ from a conversation with a doctor?

[Probe: Again, we try to get any discrepancies between lay and expert knowledge and how the gap affect their understanding and risk perception]

**2. Subjective Intepretation of how T2D may impact their lives**

*Now I would like to talk about how you see yourself in terms of risk of diabetes in the future.*

1. How do you feel about having a disease that could be with you, and have to be managed, for the remainder of your life?

[Note: There will be different views between those with chronic disease and those without it]

- 1. What are some positive and negative ways your life would change if you had a chronic disease?

[Probe: Ask for specific scenarios, can be emotional, mental, physical, etc]

If you were diagnosed with diabetes, what would be the gains and losses that concern you about this condition?

- 1. What is important to you that diabetes might prevent you from doing?

(o*nce you become sick, what activities will you fear missing out on)*

[Probe: What can you do now? What might you not be able to do if you had diabetes? What could you do about it?]

[Probe: Ask for specific scenarios, can be emotional, mental, physical, etc]

1. Using the scale 0-10, what do you think is the likelihood of you getting diabetes in the near future? Suppose 0 means ‘not a chance’ and 10 means ‘100% chance’, what score would it be?

[Probe: Let’s see if 0-10 scale works. If so, we will use it again in the last section – what would you need to do to lower down the score?]

10.1 Why do you think that you are at such a risk of getting the disease?

[Probe: family history, work and living environmental factors, individual behaviours]

**3. Facilitators and Barriers of Practising T2D Preventative Behaviours**

*Thank you so much for sharing your thoughts. I would like us to talk more about healthy practices.*

1. What are you currently doing that you think is a healthy habit?

11.1 How did you get started on this? What helps you continue this habit?

1. What are you currently doing that you think is an unhealthy habit?
   1. How do you feel about having to change your lifestyle?
2. You said that your likelihood to having diabetes is X (from question 9) – What do you think you need to do lower your score? Why do you think you have not done it yet?

[Probe: Here we are trying to differentiate efficacy vs self-efficacy]

- 1. How difficult or easy is it for you to do [that behaviour]?
  2. Why is it important for you to do these things?

[Probe: to ensure high quality of life, or to live longer; what do you think will happen if you don’t do [x behaviour]?]

- 1. If you did this, apart from preventing diabetes, what other benefits might you experience.

[Examples: (1) If I lose weight, I might be more confidnt and feel more attractive; (2) Exercise might allow me to expand my social circle or it might prevent dementia; (3) If I prevent disease and reduce healthcare costs in the future, I might have more money for my kids education, or to travel]

1. What are some personal barriers that may be stopping you from starting or practicing these behaviours? How do you think they can be addressed?

[Probe: competing priorities, emotional hurdle, indifference]

- 1. What about your home or work environments? How do they help you to practice healthy habits?

- 1. What about your cultural practices? How do they make it harder or easier for you to make healthier choices?

[Probe: some elements in their culture that encourage healthy practices or unhealthy practices]

1. Thinking about all these challenges, what additional things would make it easier or motivate you?

[Probe: Ask them to walk you through an ideal situation]

1. How would you feel if you were told that your risk of diabetes is lower than you thought (Link to score they used in Q9)?
   1. Will that change your habits in any way?
   2. How about if your risk is higher than you thought (Link to score they used in Q9)?

[Probe: Elaborate on their reaction, and ask for why they would change or not change their behaviour? Does it make you feel more empowered or helpless over your body and health?]

- 1. . How do the different numbers (0-10), as your risk scoare make you feel?

[Probe: Here we trying to understand how the scales influence their motivation to change behaviour. Assess if this translates to percentages (10% vs 80% as well)

**Debrief**

Thank you for your participation in this interview. We appreciate the time you took to talk with us. Do you have any questions about the interview or the study that I can answer?

How was the interview experience for you? Was there anything that made you uncomfortable or feels offended in any way?

Is there anything you would like to tell me that was not covered in the interview? Are there any questions that you would like me to ask in future interviews, or is there anything that you would want to know about regarding this topic – what we have discussed?
